# Microfluidics-based immunofluorescence for fast staining of ALK in lung adenocarcinoma

**DOI:** 10.1186/s13000-018-0757-1

**Published:** 2018-10-16

**Authors:** Saška Brajkovic, Benjamin Pelz, Maria-Giuseppina Procopio, Anne-Laure Leblond, Grégoire Repond, Ariane Schaub-Clerigué, Diego G Dupouy, Alex Soltermann

**Affiliations:** 1 Lunaphore Technologies SA, EPFL Innovation Park-Building A, CH-1015 Lausanne, Switzerland; 2 Microsystems Laboratory 2, Swiss Federal Institute of Technology, CH-1015 Lausanne, Switzerland; 3 Institute of Pathology and Molecular Pathology, University Hospital Zurich, CH-8091 Zurich, Switzerland

**Keywords:** Microfluidic tissue processor, Immunofluorescence, Anaplastic lymphoma kinase, Lung adenocarcinoma

## Abstract

**Background:**

Anaplastic lymphoma kinase (*ALK*) is a key oncogenic driver in lung adenocarcinoma patients and its fusion proteins are routinely assessed. The microfluidic tissue processor (MTP) device is based on a chip-confined low-volume technology allowing for rapid immunohistochemistry/immunofluorescence (IHC/IF) stainings of formalin-fixed paraffin-embedded (FFPE) or frozen tissue samples.

**Methods:**

A novel ALK IF protocol was developed for the MTP device using the primary mouse anti-human ALK antibody clone 5A4. FFPE tumor whole sections from 14 resected lung adenocarcinoma patients documented to be ALK positive (ALK+) by automated chromogenic IHC and/or FISH were used. MTP-derived IF immunoreactivity was measured by computerized analysis of digitalized images on individual frames of tumor epithelia and surrounding stroma, using an ImageJ plug-in.

**Results:**

The 5A4 antibody yielded saturated immunoreactivity at an incubation time of 4 min on a titration curve ranging from 2 to 32 min. Total staining time on the MTP device was 18 min including secondary IgG Alexa Fluor 647. MTP-based ALK IF confirmed all 12 cases; with epithelial signal above stromal staining based on computerized pixel-based measurement. MTP-IF (mean intensity levels 458 to 1301) and chromogenic IHC (H-score 120 to 300) showed an equal range of variation of 2.8 and 2.5 folds, respectively, and a trend for direct correlation (*p*-value 0.051).

**Conclusion:**

The newly developed protocol for immunofluorescent detection of ALK protein with the MTP device confirms chromogenic IHC results on FFPE lung adenocarcinoma specimens. MTP-based IF is fast and reliable. We foresee this study to be a first step opening the road for further realization of microfluidic-based assays for rapid simultaneous detection of targetable oncogenic and immune-system related markers in their topographical context to investigate tumour heterogeneity and micro-environmental interactions.

**Electronic supplementary material:**

The online version of this article (10.1186/s13000-018-0757-1) contains supplementary material, which is available to authorized users.

## Background

Rearrangement of the anaplastic lymphoma kinase (*ALK*) gene is an oncogenic driver event typically occurring in young non-smokers suffering from a *KRAS/EGFR* wild-type lung adenocarcinoma [1, 2]. A paracentric inversion within the *ALK* gene on chromosome 2p23 fuses with the *EML4* gene or other partners. This chromosomal rearrangement results in cytosolic expression of an oncogenic fusion protein, which are targeted by ALK tyrosine kinase inhibitors (TKI), such as crizotinib and lorlatinib. Nevertheless, TKI resistance can occur via development of *ALK* mutations. The *ALK* resistance mutation L1198F induced by lorlatinib, however resensitize tumor cells to crizotinib [3]. Thus, a suitable sequence of targeted and classical chemotherapy may result in long lasting patient survival.

For the prescription of such regimens, the reliable assessment of ALK protein expression and its alteration on sequential tumor biopsies together with corresponding genomic data is of high importance. The four main methods for ALK assessment include chromogenic immunohistochemistry (IHC), fluorescence in-situ hybridization (FISH), reverse transcriptase polymerase chain reaction (RT-PCR) and next generation sequencing (NGS). The *ALK* status is routinely assessed in diagnostic surgical pathology by either or a combination of the four methods. Yet, there is no international gold standard for the best assay as all four methods have their respective pros and cons in terms of pre- and analytical process turnaround time, accuracy and cost [4, 5]. Regarding IHC, *ALK* rearrangements are detected regardless of variant and fusion partner. Despite the initial poor performances of ALK on lung adenocarcinoma, immunohistochemistry is currently considered reliable and cheap, due to employment of signal amplification system and optimized anti-ALK antibodies, such as clone 5A4 (Novocastra) or D5F3 (Ventana), with the latter being approved by FDA (Food and Drug Administration) as companion diagnostic test for *ALK* rearrangements [6].

Microfluidic-based IHC/IF using devices such as the microfluidic tissue processor (MTP) enhances quality and reproducibility of immunoreactivity [7]. The MTP creates a 100 μm high incubation chamber above the tissue covered by a microfluidic chip. Fast fluidic exchange (FFEX) and border gaskets allow for low incubation times due to rapid paratope-epitope interaction and homogenous staining across the zone of confinement with sharp edges. Using this technology, a better repartition of HER2 negative versus positive breast carcinomas was achieved, resulting in a 90% decrease of ambiguous results. Furthermore, the level of HER2 protein expression, as continuously quantified using microfluidic precision IF, predicted the number of *HER2* gene copies obtained from FISH [8].

Immunofluorescence is a valid and in e.g. nephron-pathology widely used alternative method to immunohistochemistry with the advantage that a 2-layered protocol of primary and labelled secondary antibody is often sufficient to obtain good signal-to-noise ratio [9]. IHC usually requires further amplification steps including hapten linkers and multimers. IF markers can also be more easily multiplexed by the tyramide signal amplification (TSA) technology [10], allowing for parallel assessment of diagnostic, oncogenic and immune system-related markers in respective topographic context.

In this study, we have developed an ALK immunofluorescence protocol for the MTP device to be performed on FFPE cancer tissue section. Except pre-treatment including deparaffinization, hydration and antigen retrieval, all incubation steps were performed on-chip within the MTP device. We used whole tumor sections from surgically resected lung adenocarcinoma patients documented to be ALK+ by chromogenic IHC and/or FISH. MTP-derived IF immunoreactivity was measured by computerized analysis of digitalized images on individual frames of tumor epithelia and surrounding stroma.

## Methods

### Patient tumor tissues

Whole section cuts of formalin-fixed, paraffin-embedded (FFPE) tumor tissue of 14 surgically resected lung adenocarcinoma patients of the University Hospital Zurich were used. One patient (Case N°11) was represented by two samples. In total, 15 surgical specimens were analyzed. Three specimens (Case N°12, 13 and 14) were used for protocol evaluation. The elaborated protocol was applied to the series of 12 other samples. As control for ALK-negative samples, we included a tissue microarray (TMA) of 196 surgically resected non-small cell lung carcinoma (NSCLC) patients. The Ethical Commission of the Canton of Zurich approved the study under reference number KEK ZH-Nr. 29–2009/14.

### Blocking solutions, primary antibodies and detection systems used for the MTP-IF

To determine the optimal blocking solution the following reagent were tested: bovine serum albumin (BSA) 1% (Life Technologies), horse serum 2.5% (Vector Laboratories), Top Block (Lubioscience) and Sudan Black B (Sigma-Aldrich).

Mouse anti-human ALK antibody clone 5A4 (Novocastra) was used with a dilution of 1/10 in PBS-Tween (PBST) 0.05% (Fischer Scientific). Mouse anti-human pan-cytokeratin (pan-CK) antibody cocktail AE1/AE3 (Dako) was diluted 1/100.

Reagents used for the detection step included the goat anti-mouse IgG (H + L) highly cross-adsorbed Alexa Fluor 647 (Life Technologies, 1/40), ImmPRESS system with horseradish peroxidase (HRP)-coupled secondary antibody (Vector Laboratories, ready-to-use) and tyramide signal amplification (TSA) kit with Alexa Fluor 647 labelled tyramide diluted 1/100 in amplification buffer according to manufacturer recommendations (Life Technologies).

For nuclear counterstaining, DAPI was included in the SlowFade Gold Antifade mounting solution (Life Technologies, RTU).

### Tissue slide pre-processing for MTP-IF

Two μm thick FFPE tumor whole sections were mounted on glass slides and manually (OFF-chip) de-paraffinized by heating at 65 °C for 10 min followed by 10 min incubation with dewaxing solution (Histoclear, National Diagnostic); after being rehydrated with decreasing concentrations of ethanol (100, 95, 70 and 40% *v*/v, Fischer Chemical) down to tap water, slides underwent heat-induced antigen retrieval step in TRIS/EDTA solution pH 9 (Dako) at 95 °C for 30 min. Slides were cooled-down for 20 min in the antigen retrieval solution, washed and kept in PBS until the staining process with the microfluidic device started.

### Microfluidic setup

**Fig. 1:**
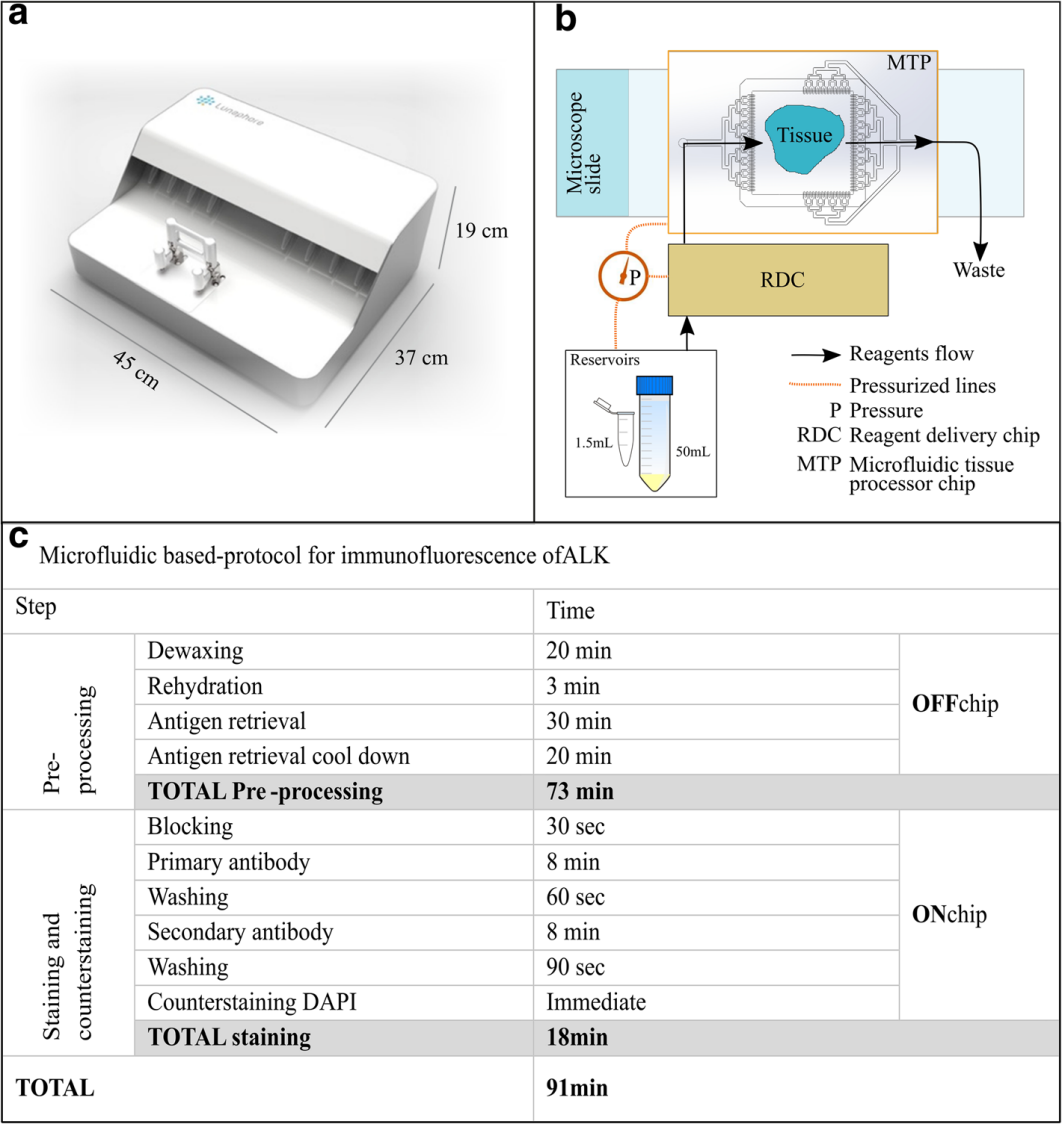
Working principle of the microfluidic tissue processor (MTP) and detailed time protocols. **a-b** The MTP device is based on a pressure-driven system, which controls the delivery of the reagents from reservoirs of either 1.5 or 50 mL. Reagents are driven to the tissue slide by passing through the reagent delivery chip (RDC) and the microfluidic tissue processor chip (MTP). The MTP chip is placed on top of the tissue section, resulting in the formation of a thin closed reaction chamber. This allows for confined epitope-antibody interaction. The exchange of reagents is done in a timeframe of 1 s, following the principle of the fast-fluidic exchange technology (FFEX). Thereafter, reagents are delivered into the waste. The clamping of the slide and the protocols details are defined via a user interface connected to the system. **c** The tissue slide pre-processing was performed manually (OFF chip) and included (i) dewaxing for 10 min at 65 °C followed by 10 min incubation with dewaxing solution, (ii) rehydration with decreasing concentrations of ethanol down to tap water, (iii) heat-induced antigen retrieval with TRIS/EDTA pH 9 at 95 °C for 30 min and (iv) cool-down for 20 min. The direct ALK IF staining was performed on the microfluidic device (ON chip) by using the primary antibody mouse anti-human ALK (Novocastra, clone 5A4) and a fluorescently-labelled secondary anti-mouse IgG antibody (Alexa Fluor 647). The blocking solution was 2.5% horse serum. DAPI was included in the mounting solution for counterstaining

Glass slides were inserted into the MTP device, which contains a clamping system that interfaces the microfluidic chip with the tissue sample via an elastomeric gasket, forming a chamber of reaction of 100 μm height. Reagents were sequentially delivered into the chamber, incubated and washed over the surface of the tissue cuts on a one-second time resolution, as previously described [7] and detailed in Fig. 1b. The reagent delivery system (RDS) consisted of a set of valves used to deliver reagents from pressurized reservoirs of 50 mL and 1.5 mL to the reaction chamber. The microfluidic design allows for rapid paratope-epitope interactions, thus short incubation times are possible for immuno-stainings. A graphical user interface installed in a computer was used to control all protocol steps.

### Immunofluorescence staining on the MTP device

For direct pan-CK IF, both primary and secondary antibodies (IgG^AF647^) were incubated for 4 min each; other protocol steps were as in Fig. 1c. Total duration of the staining was 10 min.

Detailed protocol and total staining time for direct ALK IF using the fluorophore-conjugated secondary antibody (IgG^AF647^) are shown in Fig. 1c.

For TSA mediated ALK indirect IF, the HRP-conjugated secondary antibody was incubated for 8 min, followed by fluorescently labelled tyramide incubation for 2 min. Other protocol steps were as detailed in Fig. 1c.

Negative controls were done by replacing the primary antibody with the antibody diluent PBST 0.05%; all other steps of the negative controls correspond to the same staining conditions of the experiment.

### ALK chromogenic IHC staining and scoring

Two μm thick sections of representative tumor blocks were used. The Refine 30/30 HRP protocol was performed on a Leica Bond-Max automated IHC platform as following: mounted tissue cuts were dewaxed in a 60 °C oven, rehydrated and boiled for 30 min for heat induced epitope retrieval (HIER) at pH 8 Tris-buffered EDTA. Primary antibody 5A4 was diluted 1/10 and incubated for 30 min at room temperature (RT). Thereafter, the Refine post-primary antibody linker was applied for 15 min at RT, followed by the Refine polymer coupled to HRP for another 15 min at RT. Diaminobenzidine (DAB) was incubated for 4 min, followed by hematoxylin for 15 min. Chromogenic immunoreactivity was H-scored, whereby whole section tumor areas with semi-quantitative intensities 0, 1, 2 or 3 were multiplied by individual frequencies of stained cells and then summed up (range 0 to 300). As previously described in the European Thoracic Oncology Platform (ETOP) ALK 001 study, a threshold H-score value higher than 120 was thereafter used to define ALK positivity [11, 12].

### ALK fluorescent in-situ hybridization

Four μm thick sections were incubated with a dual color break apart FISH probe for chromosome 2p23 (LSI 3’-*ALK* Spectrum-Orange and 5’-*ALK* Spectrum-Green, Vysis/Abbott Molecular) according to the manufacturer’s protocol. For each case, 100 non-overlapping nuclei were evaluated using a fluorescence microscope (Zeiss Axioskop) with a 100-fold magnification oil objective. Z-stacks of 20 images with 0.5 μm step distance were merged. Positive *ALK* FISH was defined as > 15% cells with either break-apart or isolated 3′ signals. Isolated 5′ signals were considered negative.

### Image acquisition

The brightfield images of the chromogenic ALK IHC stainings were digitalized on a NanoZoomer Digital Pathology scanner (Hamamatsu, Japan). For IF we used a Zeiss AxioImager M2 m microscope and an Olympus slide scanner VS120-L100. Each image was taken in both DAPI (Alexa350) and Cy5 (Alexa647) channels. For all images within a figure, the same parameters of acquisition, such as filter set, exposure time, and filter intensities were used.

### Image analysis

Image quantification was done using custom written ImageJ macros. For quantification of the IF signal, 25 representative regions of interest (ROI) were selected both on tumor epithelia and on stroma areas. For the tumor epithelia ROIs, which contain both ALK positive and negative areas, the pixels containing positive signals were selected in ImageJ by the Huang automatic thresholding before recording the mean fluorescence intensity. For the stroma areas, where the un-specific signal is reported to appear is various structures, the mean fluorescence intensity of whole ROI was recorded, independently of the expression level and cell type to include fluorescent signal of any origin. The average signal and corresponding standard deviation for tumor epithelia and stroma areas were calculated by averaging the mean intensities from all 25 ROIs.

### Statistical analysis

MTP-derived IF values were correlated with chromogenic IHC and break-apart FISH using the Kendall’s tau-b test. A *p*-value of ≤0.05 was considered significant.

## Results

### Determination of optimal blocking solution and detection system for the MTP-based IF

A schematic depiction of the MTP device with its working principle is shown in Fig. 1a-b. The tissue slides pre-processing and staining protocol steps for the MTP device are shown in Fig. 1c.

Different blocking solutions were tested (Additional file 1: Figure S1). The optimum blocking solution allowing for a good specific signal with minimal non-specific background turned out to be 2.5% horse serum. Regarding the detection system, two methods were tested: fluorophore-conjugated secondary antibody IgG^AF647^ versus tyramide signal amplification HRP-TSA^AF647^ (Additional file 1: Figure S2). Both methods resulted in strong and fast ALK detection. However, we inferred that the enzyme-based approach has less controlled reaction kinetics and might cause non-linearity. Therefore, the IgG^AF647^ secondary antibody was further used due to the aim of performing ALK quantification. Further, we evaluated the border of confinement delimitated by the gasket. The gasket allowed for sharp transition of AF647 positive immunoreactive tumor areas to unstained areas under or outside the rubber sealing that present only nuclear counterstaining due to addition of DAPI to the final mounting solution (Additional file 1: Figure S3).

### MTP-based IF staining allows for short antibody incubation times

Pan-cytokeratin (pan-CK) is widely used in diagnostic pathology to identify tumor epithelial cells. As the lung adenocarcinoma samples used in this study are of epithelial origin, the first test performed with the MTP device was a direct IF for pan-CK as a positive control staining. Pan-CK was specifically and rapidly detected in the cytoplasm of epithelial cells (Additional file 1: Figure S4).

**Fig. 2:**
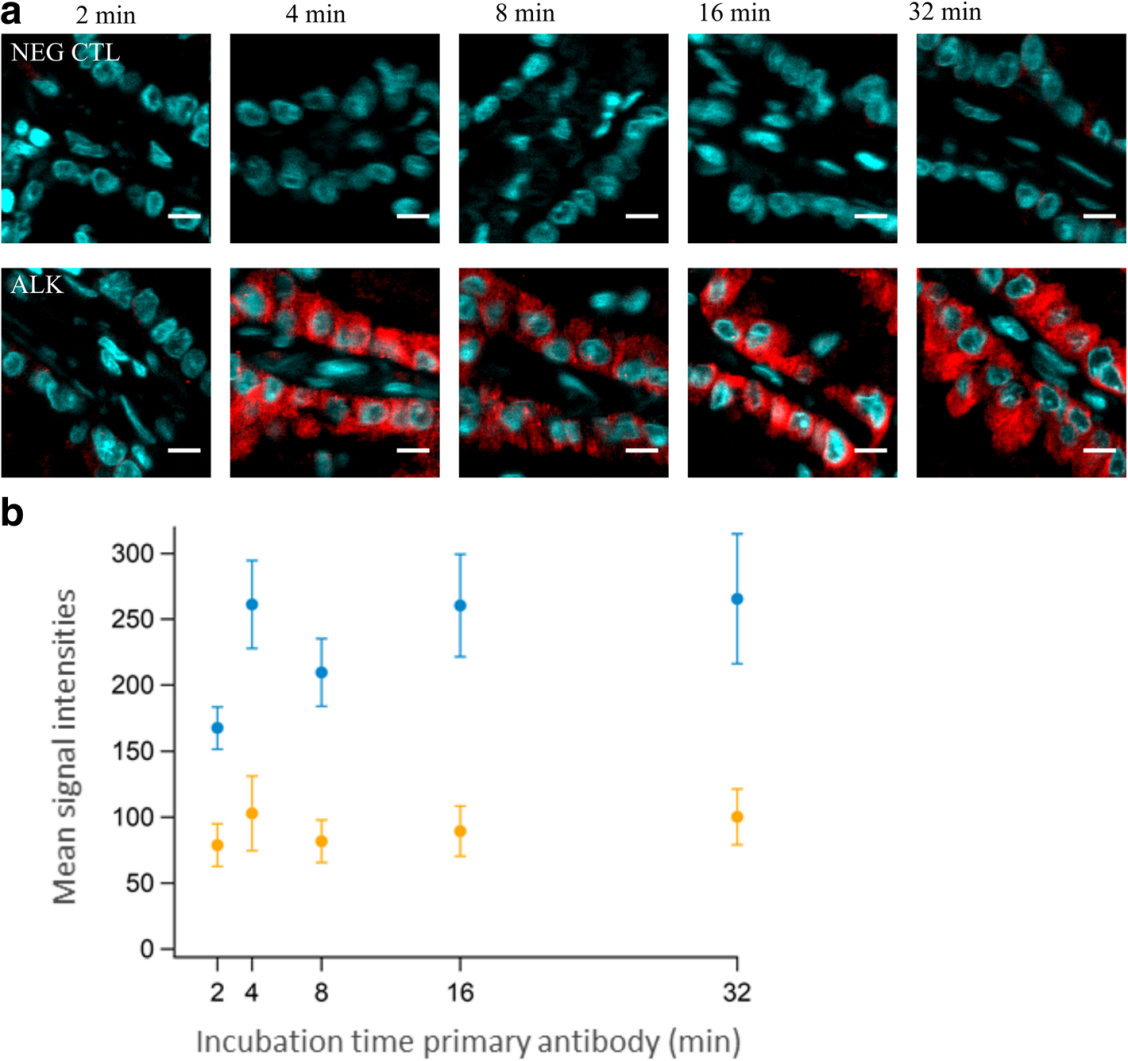
Determination of optimal ALK primary antibody incubation time. **a** Tissue cuts (Case N°12) were incubated for 2, 4, 8, 16, or 32 min either with PBST 0.05% (CTL NEG, first row) or the anti-human ALK antibody (ALK, second row). The incubation time of the secondary antibody was kept constant at 8 min. The same region of interest (ROI) is presented for ALK IF and its negative control. Scale bar is 10 μm. **b** Quantification of the ALK stained slides shown in (**a**). For each incubation time, 25 ROIs were manually placed in areas of ALK+ tumor epithelia and in areas of stroma only. The graph shows the average of the mean intensities of all 25 ROIs for ALK+ tumor epithelia (blue) and stroma (orange) with corresponding standard deviations

In order to define the optimum primary antibody incubation time for ALK, serial sections of lung adenocarcinomas were incubated with anti-human ALK antibody clone 5A4 for 2, 4, 8, 16, or 32 min (Fig. 2a, lower panels), while the incubation time of the secondary antibody was kept constant at 8 min. For each condition a respective negative control was performed; no ALK specific signal was found in the negative controls (Fig. 2a**,** upper panels). As shown on the images and the plot, a strong signal intensity for ALK protein in the cytosol of tumor epithelia was reached already after 4 min of primary antibody incubation. Longer primary antibody incubations did not lead to a further increase of signal intensity (Fig. 2b). We noted a good separation between ALK specific signal on tumor epithelia and stromal background.

### Tumor epithelia-associated ALK staining on lung adenocarcinoma cases

**Fig. 3:**
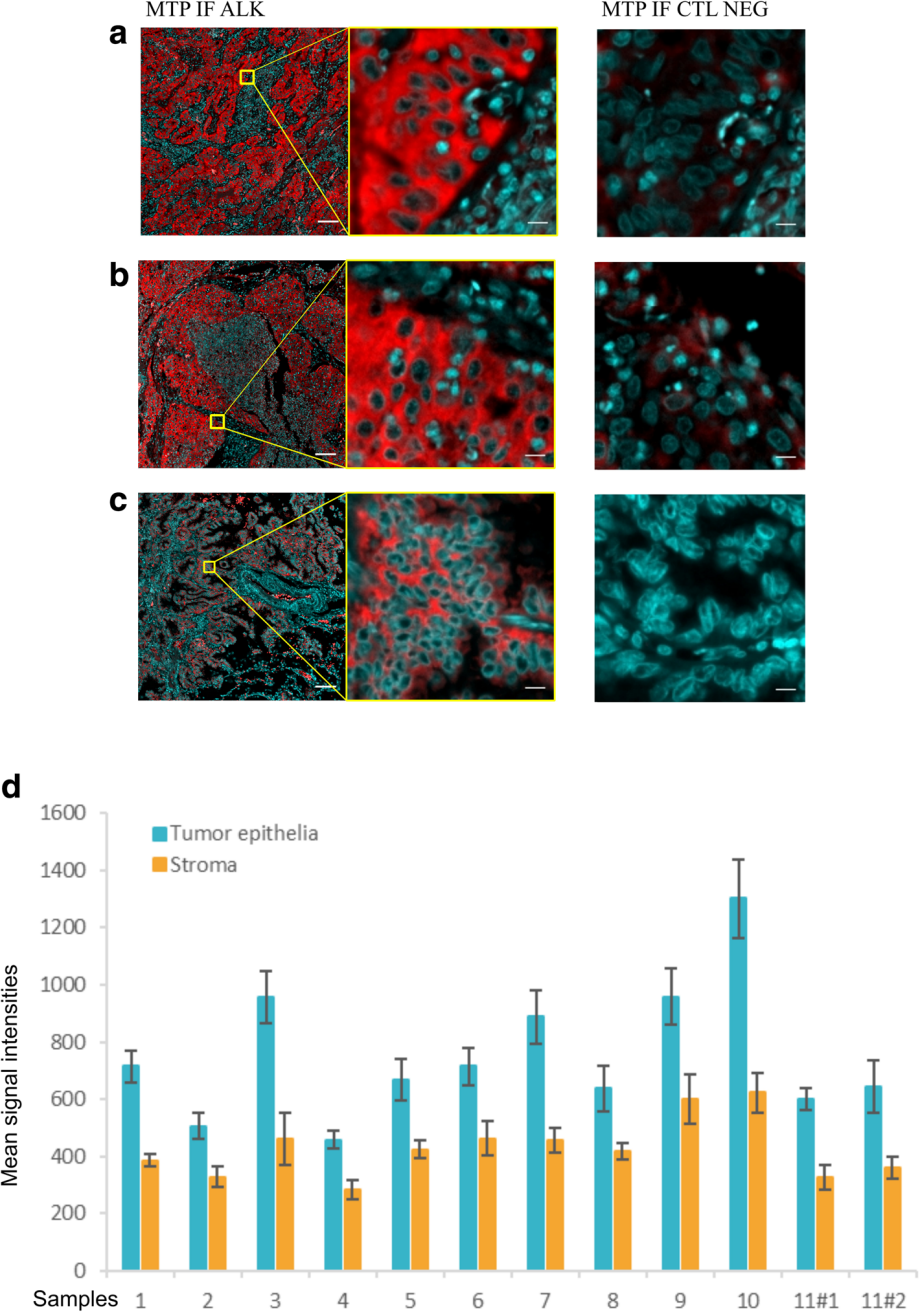
Variation of ALK immunoreactivity in a series of 12 lung adenocarcinomas. Three representative cases of microfluidic-based ALK IF (MTP IF ALK) are shown in **a** Case N°10, **b** Case N°7 and **c** Case N°3. Scale bars are 100 μm and 10 μm for the first and second column, respectively. The third column represents the negative control (CTL NEG) where PBST 0.05% was used instead of the primary antibody. The shown region of interest for the negative control is the same as for ALK IF; scale bar is 10 μm. **d** Quantification of the ALK IF signals. For each case, 25 ROIs were manually placed in both ALK+ tumor epithelia and stroma regions. The graph shows the average of the mean intensities of all 25 ROIs for tumor epithelia (blue) and stroma (orange) with corresponding standard deviations. Case N°11 is present twice (11#1 and 11#2) as two samples with distinct localization (lung primary and parietal pleura, respectively) were obtained from the same patient

A comparison of the relative signal intensity for the microfluidic-based ALK IF between areas of tumor epithelia and adjacent stroma in three representative cases of lung ADC is shown Fig. 3a-c. In all 12 surgical specimens, the ALK specific signal was detected on tumor epithelia above the stromal background intensity (Fig. 3d). The ALK IF expression was also visually compared to the corresponding chromogenic IHC for each case, showing matching regions of ALK positivity (see Additional file 1: Figure S5 for a representative case).

**Fig. 4:**
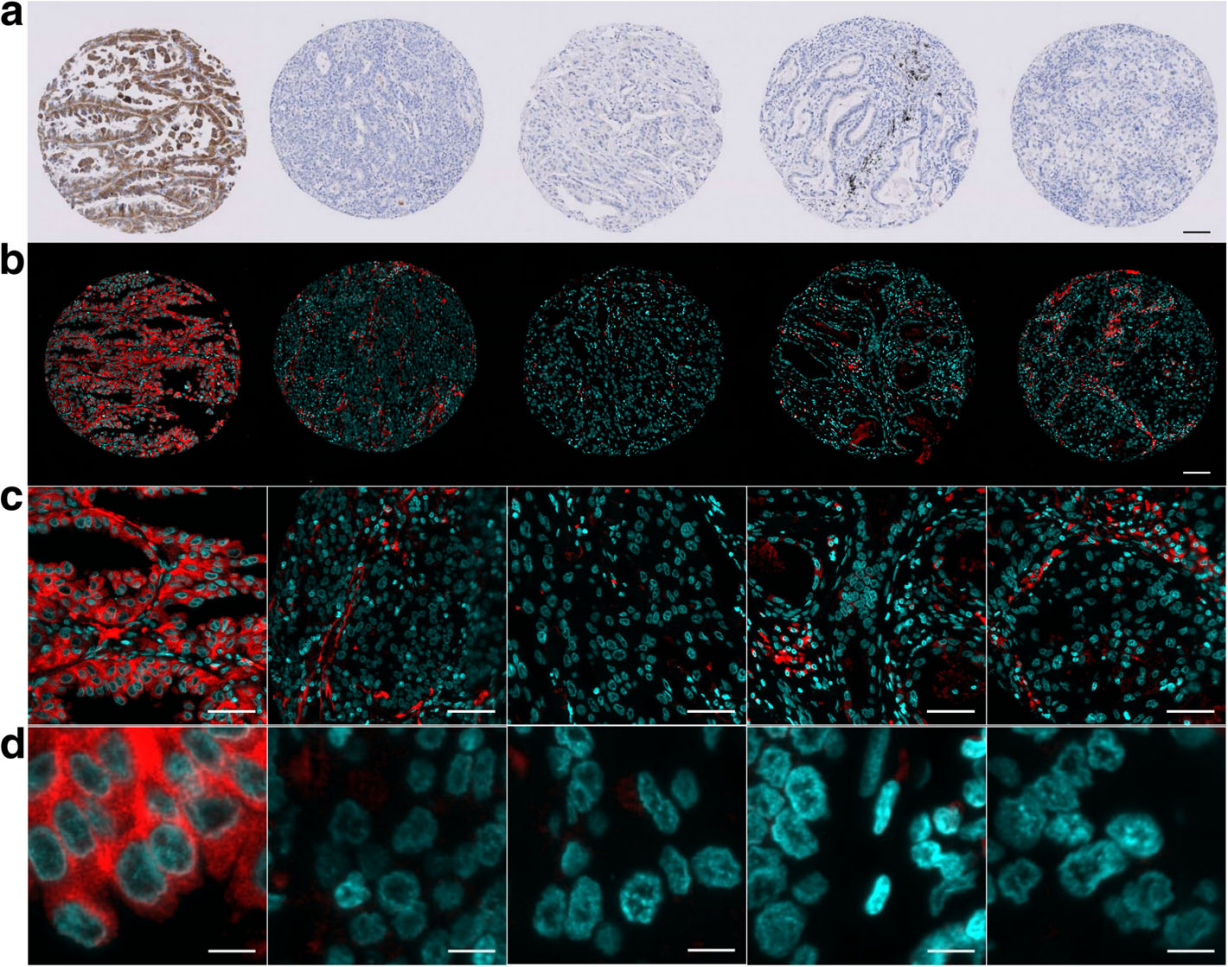
ALK MTP-IF on five lung ADC from a NSCLC TMA. **a** Shown are five tumor cores including one ALK-positive case next to four ALK-negative cases (left to right) as assessed by chromogenic IHC; the black coloration visible on the fourth core is anthracosis. The same cores as in (**a**) have been stained for ALK by MTP-based IF (**b**-**d**) employing the same protocol as for the ALK-positive ADC cases in Fig. 3. Scale bar 100 μm (**a**-**b**), 50 μm (**c**) and 10 μm (**d**)

The same MTP-based ALK IF protocol was used for a NSCLC TMA. Five lung ADC tumor cores are shown, including one ALK-positive and four ALK-negative cases, as assessed by IHC (Fig. 4a). Compared to the ALK-positive core, no specific ALK signal was detectable in the tumor epithelia of the four negative cases; as reported earlier, un-specific signal was detectable in the stromal compartment, like the intra-alveolar macrophages (Fig. 4b-d).

### Correlation of IF values with chromogenic IHC and break-apart FISH

**Fig. 5:**
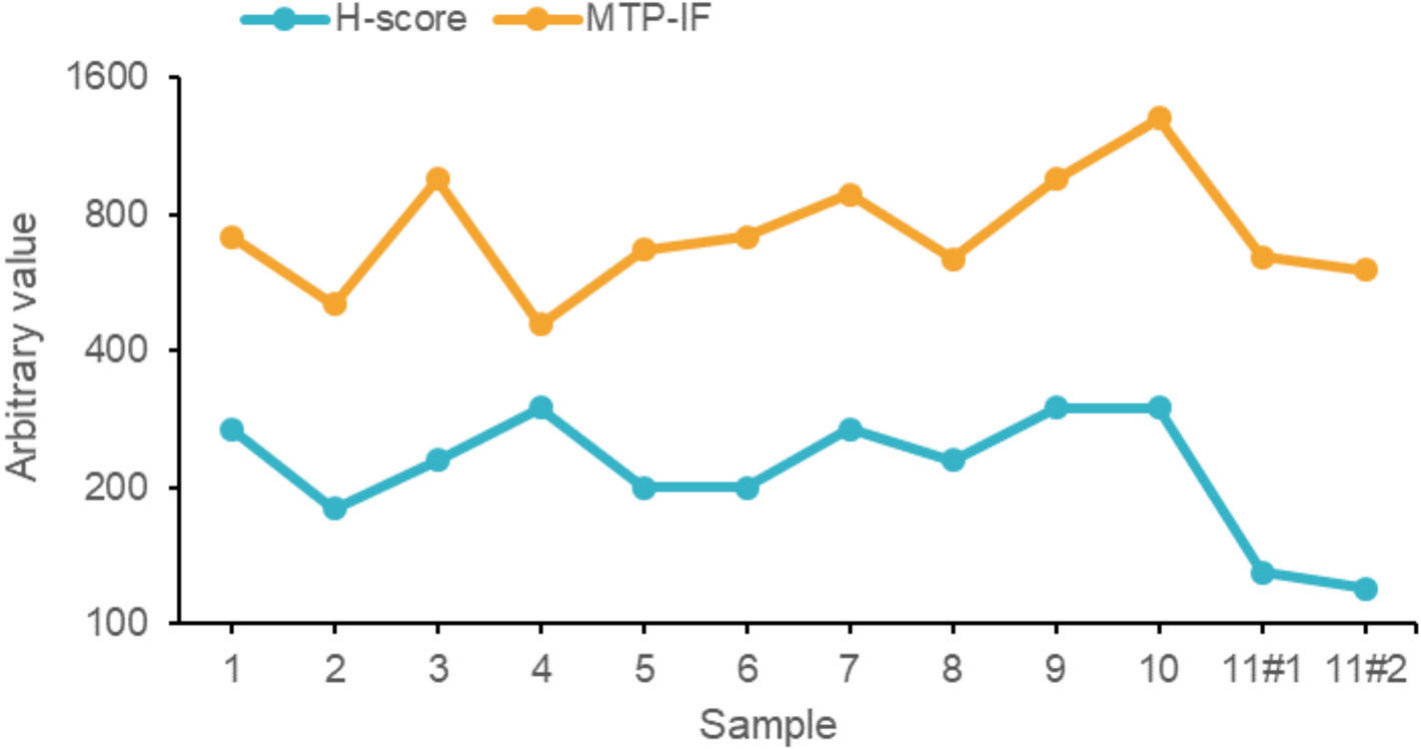
Correlation of the ALK MTP-IF values with chromogenic IHC. Graphical visualisation of the “H-score” (blue) and “MTP-IF” (orange) values for ALK staining on 12 ALK-positive lung ADC. Sample 11#1 and 11#2 represent two specimens with distinct localization (lung primary and parietal pleura, respectively) from the same patient (Case N°11)

A threshold H-score value of 120 was previously defined for ALK positivity [11, 12]; the ALK+ cases included in this study had a chromogenic ALK immunoreactivity of H-score ≥ 120 up to 300 (Additional file 2: Table S1). All 12 ALK+ samples analysed by MTP-IF were concordant with chromogenic IHC. Chromogenic IHC of ALK+ cases showed a variation of 2.5-fold regarding H-score level (120 to 300). Interestingly, a similar dynamic range with 2.8-fold variation was found for MTP-IF mean intensity levels (458 to 1301) (Fig. 5). When correlating these values, we found a nearly statistical significance for chromogenic IHC with MTP-IF (*p*-value 0.051) but not with FISH (*p*-values > 0.2).

For case N°4, the *ALK* status was discordantly assessed by IHC (positive: H-Score 300) and FISH (negative: break apart found in 10% of tumor cells). This same case showed an opposite trend when comparing microfluidic IF with IHC results: although exerting the highest score by IHC, it resulted in the lowest mean intensity of the whole series by MTP-IF (value = 458), thus better correlating with the negative FISH result than the H-score.

## Discussion

In this study, we have used the novel technology of microfluidic-based immunofluorescence for the assessment of *ALK* status in lung adenocarcinoma patients, using FFPE tumor tissue whole sections from surgical specimens. We were able to detect specific ALK immunoreactivity on tumor epithelia of lung adenocarcinoma in a fast, reliable, and automated manner, confirming the IHC results obtained by routinely used staining procedures.

In some area of the tissue, the non-specific signal of the 5A4 antibody was present in the stroma on both IHC and IF stainings. The non-specific activity of the 5A4 antibody is well documented by the supplier as a diffuse appearance, but also sporadic staining of connective tissue, fibroblasts, necrotic/degenerated cells or alveolar macrophages.

The ALK staining resulting from the protocol developed in IF correlates with the one performed by standard IHC. Firstly, visual comparison of the same region of interest between IHC and IF, showed the same foci of heterogeneous ALK expression between the two techniques. Secondly, the computerized analyses of the tumor specific ALK signal obtained with the MTP-IF correlated with the H-score values attributed to the IHC stainings.

Sample nr. 4 was the only case where the correlation between H-score and MTP-IF did not follow the same trend: although scored with the highest mark by IHC, it exerted the lowest score by MTP-IF. Interestingly, it was scored as negative by *ALK* break-apart FISH. Furthermore, *ALK* status for the same sample was assessed by HTG EdgeSeq ALKPlus assay, an in vitro diagnostic NGS-based assay intended to measure mRNA ALK gene fusion events in FFPE NSCLC specimens. Remarkably, *ALK* rearrangement status was reported to be negative (data not shown), suggesting a closer correlation of MTP-IF to FISH and NGS results as compared to IHC for this peculiar case.

Alternative to binary scoring, an intensity-based quaternary scoring system (0–3+) was suggested for ALK IHC, similarly to HER2 (human epidermal growth factor receptor 2) diagnostic test in breast cancer [13, 14]. Although high correlation was reported for ALK FISH and IHC (5A4) scored as 0–1+ (FISH-negative) and 3+ (FISH-positive), the 2+ scored cases were variable with equal repartition in FISH-positive and negative [15]. We previously showed the MTP-based HER2 staining to allow for better positive/negative separation among the 2+ ambiguous cases as assessed by the FISH [7, 8]. In a similar manner, the ALK MTP-IF may better correlate with the *ALK* status as assessed by FISH. Testing of such hypothesis will require further investigation.

Microfluidics is a powerful novel technology in the field of tissue diagnosis [16–18], but only a few instruments are commercially available. The fast-fluidic exchange (FFEX) technology within the MTP device allows for a rapid and controlled exchange of reagents while exposing the tissue surface uniformly to the reagents. This allows the primary antibody to rapidly reach its epitope on the targeted protein and to be detected by the secondary antibody. In this study, using the MTP technology, we show that the signal of ALK IF saturates after 4 min of primary antibody incubation. The reaction taking place in the MTP chamber is highly controlled, and the borders limiting the tissue area exposed to the reagents can be visualised if the tissue is present inside and outside of the chamber. The gasket that allows the closing of the chamber delimits the reagent distribution and thus serves as an internal control for staining quality and tissue auto-fluorescence. Moreover, we have addressed the issue of auto-fluorescent lung structures by analyzing specific immunofluorescent ALK signals on tumor epithelia and on adjacent stroma on multiple individual frames laid over the tumoral structures on whole sections.

One further advantage of the MTP device is its open-system design, meaning that any kind of reagents compatible with the internal surfaces of device can be used. E.g. ALK immunoreactivity could be visualized by two techniques of detection: [1] a direct staining method using a secondary antibody labelled with the fluorophore AF647; [2] an amplification-based staining method, where the HRP-conjugated secondary antibody catalyses a fluorescent (AF647) tyramide-based signal amplification (TSA). Direct immunofluorescence has the advantage of being potentially quantitative due to direct probing of the epitope-specific antibody by secondary IgG coupled to a fluorophore, while having the disadvantage of generally low signal to noise ratio due to lack of amplification steps. Nevertheless, both detection systems gave a specific and strong ALK signal.

Of particular interest is the question of how diagnostic, predictive oncogenic and immune-therapy related markers are correlated among each other in relation to topographical proximity and tumor heterogeneity, respectively. Research has therefore focused on implementation of multiplexed immunofluorescence methods, using e.g. the TSA technology. A multi-parametric immunofluorescence study including ALK, CD8, PD-1 and PD-L1 was recently performed. In comparison to EGFR-mutated or WT lung adenocarcinomas, ALK-positive tumors had a higher expression of PD-L1 and a higher number of intra-tumoral CD8+ T cells or PD-1+ CD8+ T cells [19].

Therefore, rapid ALK immunofluorescence allowing for combined detection of multiple markers is likely to be highly exploited in a near future.

## Conclusions

In summary, we report here the first MTP-based IF protocol for assessment of ALK protein expression in FFPE tumor tissue of lung adenocarcinoma patients. We foresee that this study is a first step that opens the road for further development of microfluidic-based assays to rapidly detect multiple markers in their topographical context to better understand tumour heterogeneity and micro-environmental interactions to advance in targeted cancer therapy.

## Additional files

Additional file 1: **Figure S1.** Evaluation of different blocking solution for ALK IF. **Figure S2.** Evaluation of two different detection systems for ALK IF. **Figure S3.** Sharp confinement of ALK immunoreactivity inside the MTP chamber. **Figure S4.** Immunofluorescence for pan-cytokeratin using the MTP device. **Figure S5**. Comparison between IF and IHC. (PDF 929 kb)

Additional file 2: **Table S1**. Summary of clinico-pathological patient data. (DOCX 79 kb)

## Abbreviations

ALK: Anaplastic lymphoma kinase
ALK+: ALK positive
CTL NEG: Control negative
DAB: Diaminobenzidine
ETOP: European thoracic oncology platform
FDA: Food and drug administration
FFEX: Fast-fluidic exchange technology
FFPE: Formalin-fixed, paraffin-embedded
FISH: Fluorescence in-situ hybridization
HER2: Human epidermal growth factor receptor 2
HIER: For heat induced epitope retrieval
HRP: Horseradish peroxidase
IF: Immunofluorescence
IHC: Immunohistochemistry
MTP: Microfluidic tissue processor chip
NGS: Next generation sequencing
NSCLC: non-small cell lung carcinoma
PBST: PBS-Tween
RDC: Reagent delivery chip
ROI: Region of interest
RT: Room temperature
RT-PCR: Reverse transcriptase polymerase chain reaction
TKI: Tyrosine kinase inhibitors
TMA: Tissue microarray
TSA: Tyramide signal amplification
