# Supplementary material for: Microfluidics-based immunofluorescence for fast staining of ALK in lung adenocarcinoma
Source: Diagn Pathol. 2018 Oct 16;13:79. doi: 10.1186/s13000-018-0757-1 (PMC6192181; doi:10.1186/s13000-018-0757-1)
Supplement: Supplementary file 1 — Figure S1. Evaluation of different blocking solution for ALK IF. Figure S2. Evaluation of two different detection systems for ALK IF. Figure S3. Sharp confinement of ALK immunoreactivity inside the MTP chamber. Figure S4. Immunofluorescence for pan-cytokeratin using the MTP device. Figure S5. Comparison between IF and IHC. (PDF 929 kb) [file 13000_2018_757_MOESM1_ESM.pdf]

Supplementary Figures

a) Neg ctl<br>2.5% HS	b) ALK<br>No block	c) ALK<br>2.5% HS	d) ALK<br>1% BSA	e) ALK<br>Top block	f) ALK<br>Sudan black

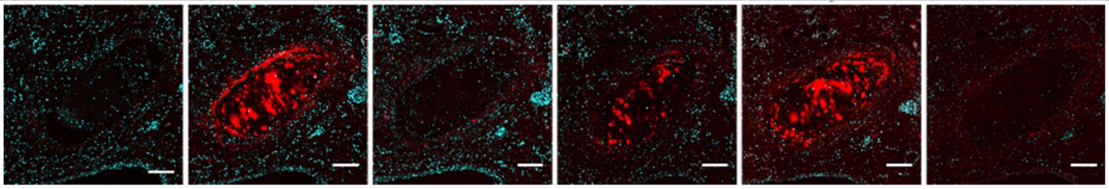

Figure S1. Evaluation of different blocking solution for ALK IF. Case N°14, showing a hyaline cartilage clasp of a lung bronchus. Unspecific staining of cartilage cells is observed with no block (b), 1% BSA (d) and Top block (e) but not with 2.5% horse serum (c). Sudan black (f) removes entire immunoreactivity. Negative control (a): no primary antibody. HS, horse serum, BSA, bovine serum albumin. Scale bar: 300 µm.

a) Leica IHC	b) MTP IF with IgG^{AF647}	c) MTP IF with HRP-TSA^{AF647}
	Ab1: 8 min, Ab2: 8 min	Ab1: 8 min, Ab2: 8 min, TSA 2min

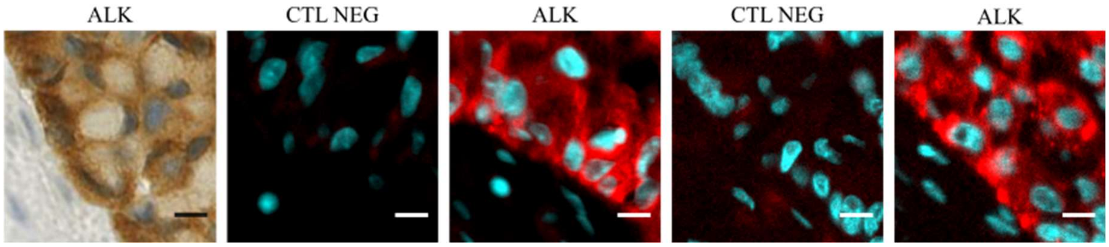

Figure S2. Evaluation of two different detection systems for ALK IF. Case N°14. Comparison between a) Leica Bond Max (chromogenic IHC) and b-c) MTP (microfluidic IF). On MTP, two protocols were used: b) secondary antibody (Ab2) IgG1 coupled to AlexaFluor (IgG^{AF647}) and c) HRP-TSA^{AF647} system. Primary antibody (Ab1) was anti-human ALK clone 5A4. Scale bar: 10 µm.

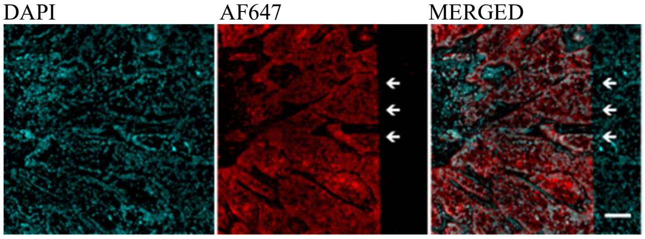

Figure S3: Sharp confinement of ALK immunoreactivity inside the MTP chamber. The rubber gasket of the MTP chip delimits a 17 x 17 mm reaction chamber. Tissue was left on purpose outside the chamber to visualize the border of reagent delivery (white arrows). DAPI was included in the mounting solution after removal of tissue section from MTP device. Scale bar: 100 µm.

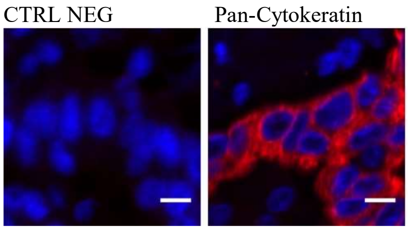

Figure S4. Immunofluorescence for pan-cytokeratin using the MTP device. Shown is pan-Cytokeratin MTP-IF (right panel) and its negative control (CTRL NEG, left panel) stained on Case N° 13. The primary and secondary antibodies were incubated for 4 minutes each. Blue, DAPI. Red, Pan-CK. Scale bar 10 μm.

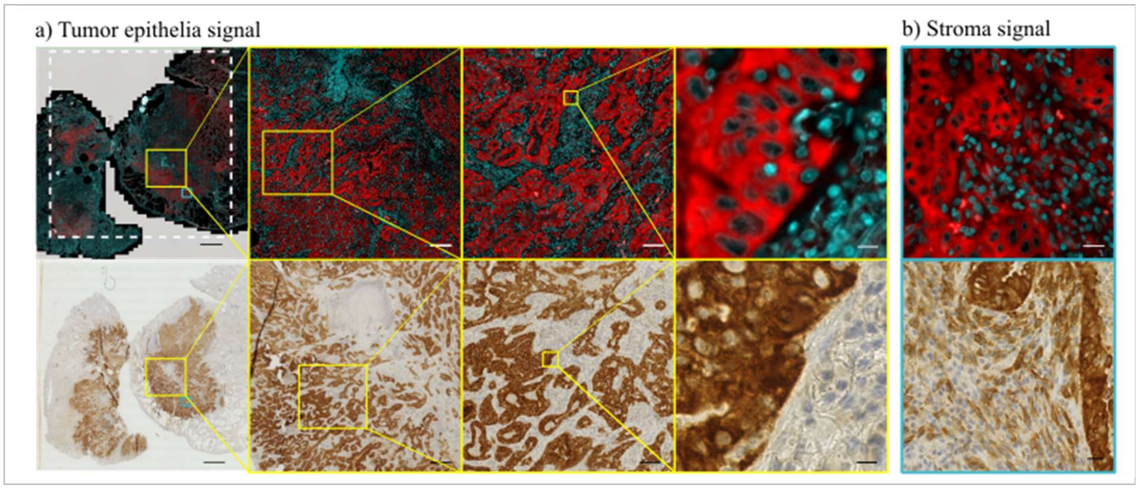

Figure S5: Comparison between IF and IHC. Case N°10. Immunofluorescence performed on MTP (first row) and chromogenic IHC on Leica Bond-Max automat (second row). Same ROI for a), strong ALK signal intensity on tumor epithelia, and b) weak to moderate signal intensity on adjacent cancer associated fibroblasts of the stroma. In a), the dashed white rectangle represents the MTP chamber and the yellow rectangle the ROI for tumor epithelia. The blue rectangle represents stroma in b). Scale bars are 2 mm, 300µm, 100µm, 10µm, and 20µm from left to right.
