# Supplementary material for: Microfluidics-based immunofluorescence for fast staining of ALK in lung adenocarcinoma
Source: Diagn Pathol. 2018 Oct 16;13:79. doi: 10.1186/s13000-018-0757-1 (PMC6192181; doi:10.1186/s13000-018-0757-1)
Supplement: Supplementary file 2 — Table S1. Summary of clinico-pathological patient data. (DOCX 79 kb) [file 13000_2018_757_MOESM2_ESM.docx]

|  |
| --- |

**Table S1. Summary of clinico-pathologic patient data.** H-score of chromogenic IHC (range 0 to 300) and % of tumor cells with positive break-apart FISH status (BA%). Case Nr°4 is FISH negative (BA10%), but strongly positive on IHC (H-score 300). G=tumor grade. TNM staging according to UICC, 8^th^ edition 2017.
